# Supplementary material for: Using macromolecular electron densities to improve the enrichment of active compounds in virtual screening
Source: Commun Chem. 2023 Aug 22;6:173. doi: 10.1038/s42004-023-00984-5 (PMC10444862; doi:10.1038/s42004-023-00984-5)
Supplement: Supplementary file 2 — Supporting Information [file 42004_2023_984_MOESM2_ESM.pdf]

# Supporting Information

# Using macromolecular electron densities to improve the enrichment of active compounds in virtual screening

*Wenzhi Ma[†1], Wei Zhang[†2,3], Yuan Le[†1], Xiaoxuan Shi[1], Qingbo Xu[1], Yang Xiao[1], Yueying Dou[1], Xiaoman Wang[1], Wenbiao Zhou[1], Wei Peng[2,3], Hongbo Zhang[*1], and Bo Huang[*1]*

1. Beijing StoneWise Technology Co Ltd., Haidian Street #15, Haidian District, Beijing 100080, China

2. State Key Laboratory of Respiratory Disease, First Affiliated Hospital of Guangzhou Medical University, Guangzhou, 510182, China.

3. Innovation Center for Pathogen Research, Guangzhou Laboratory, Guangzhou, 510320, China.

[†]Equal contributors

*Corresponding authors:

Bo Huang − Beijing StoneWise Technology Co Ltd., Beijing 100080, China;

orcid.org/0000-0003-3822-9110; Email: huangbo@stonewise.cn

Hongbo Zhang − Beijing StoneWise Technology Co Ltd., Beijing 100080, China;

orcid.org/0009-0005-1780-1968; Email: zhanghongbo@stonewise.cn

# List of Supplementary figures and tables


*Figure S1. Comparison of ExptGMS with benchmark technologies on DUD-E dataset by target class*

*Table S1. List of targets in training set and test set ($N_{training}$=73, $N_{test}$=12)*

*Table S2. Top 24 compounds selected by combining ExptGMS and GlideSP*

*Table S3. Top 24 compounds selected by GlideSP*

*Figure S2. Structures of top 24 compounds selected by ExptGMS+GlideSP*

*Figure S3. Structures of top 24 compounds selected by GlideSP*

*Table S4. Details of GBDT training parameters*

*Figure S4. Training curve of GBDT (GlideSP and multi-resolution ExptGMS)*

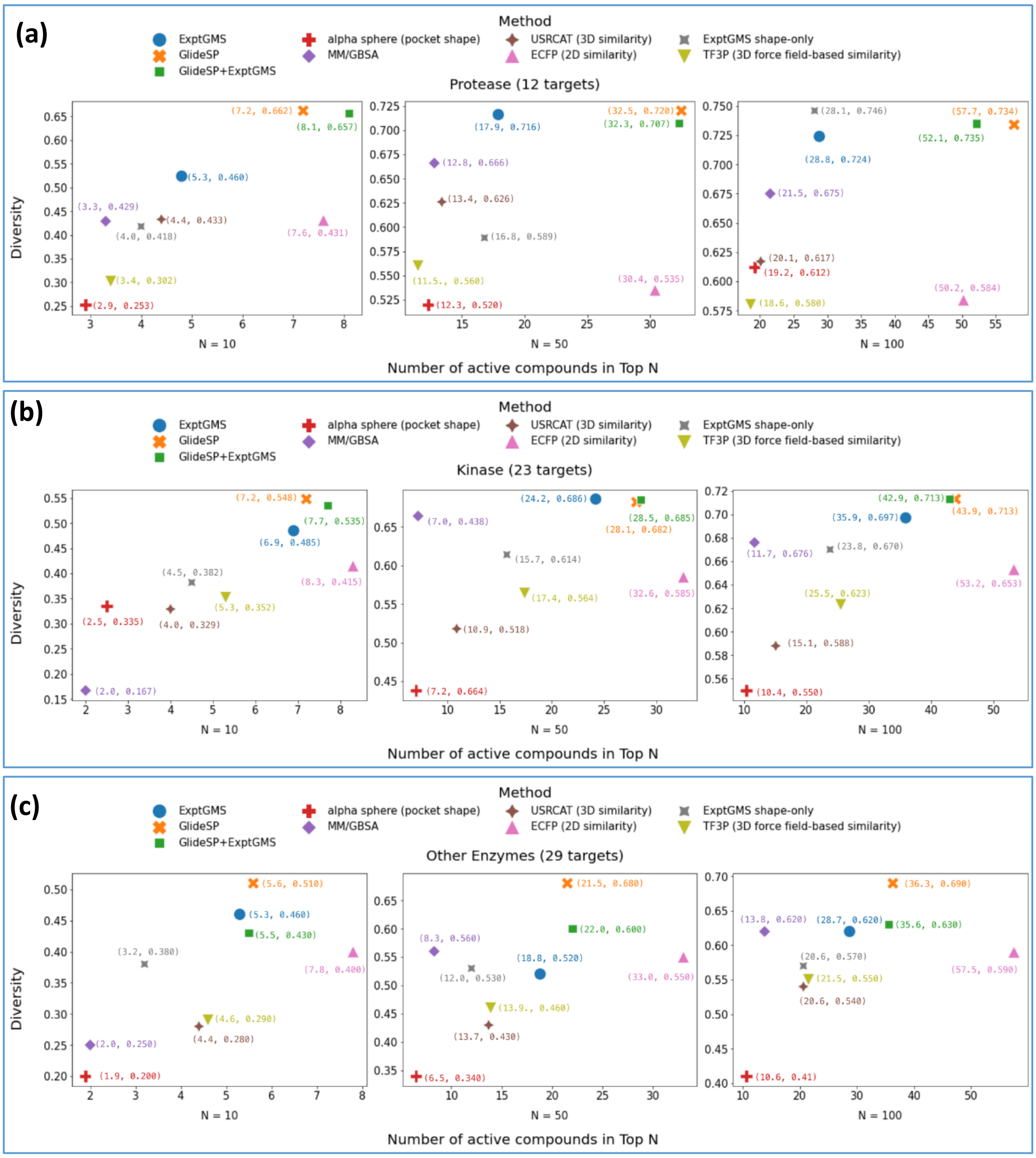


Figure S1. *Comparison of ExptGMS with benchmark technologies on DUD-E dataset by target class.* The average of pairwise Tanimoto similarities over ECFP4 fingerprints is shown for the active molecules ranked in Top N (N=10, 50, and 100) [Diversity = 1 – (average pairwise 2D similarity among the molecules)]. The 85 samples from DUD-E database used in this study can be divided into 8 datasets based on the target classes documented in the original DUD-E paper (ref). Given the limited number of targets for ion channel (N=1), cytochrome P450 (N=2), GPCR (N=5), miscellaneous (N=5), and nuclear receptor (N=8), we conducted enrichment and diversity analysis for protease (N=12), kinase (N=23), and other enzymes (N=29) and displayed the results in panel (a), (b), and (c), respectively.

**Table S1. List of targets in training set and test set ($N_{training}$=73, $N_{test}$=12)**

| Target | PDBID | Training or Test | Type |
|---|---|---|---|
| AA2AR | 3EML | Training | GPCR |
| ABL1 | 2HZI | Training | Kinase |
| ACE | 3BKL | Training | Protease |
| ACES | ‘1E66 | Training | Other Enzymes |
| ADA | 2E1W | Training | Other Enzymes |
| ADRB1 | 2VT4 | Training | GPCR |
| ADRB2 | 3NY8 | Training | GPCR |
| AKT1 | 3CQW | Training | Kinase |
| AKT2 | 3D0E | Training | Kinase |
| ALDR | 2HV5 | Training | Other Enzymes |
| ANDR | 2AM9 | Training | Nuclear Receptor |
| BRAF | 3D4Q | Training | Kinase |
| CAH2 | 1BCD | Training | Other Enzymes |
| CASP3 | 2CNK | Training | Protease |
| CDK2 | 1H00 | Training | Kinase |
| CP2C9 | 1R9O | Training | Cytochrome P450 |
| CP3A4 | 3NXU | Training | Cytochrome P450 |
| CSF1R | 3KRJ | Training | Kinase |
| CXCR4 | 3ODU | Training | GPCR |
| DRD3 | 3PBL | Training | GPCR |
| DYR | 3NXO | Training | Other Enzymes |
| EGFR | 2RGP | Training | Kinase |
| ESR1 | 1SJ0 | Training | Nuclear Receptor |
| ESR2 | 2FSZ | Training | Nuclear Receptor |
| FA10 | 3KL6 | Training | Protease |
| FA7 | 1W7X | Training | Protease |
| FABP4 | 2NNQ | Training | Miscellaneous |
| FAK1 | 3BZ3 | Training | Kinase |
| GCR | 3BQD | Training | Nuclear Receptor |
| GLCM | 2V3F | Training | Other Enzymes |
| GRIA2 | 3KGC | Training | Ion Channel |
| HDAC2 | 3MAX | Training | Other Enzymes |
| HDAC8 | 3F07 | Training | Other Enzymes |
| HIVINT | 3NF7 | Training | Other Enzymes |
| HIVPR | 1XL2 | Training | Protease |
| HIVRT | 3LAN | Training | Other Enzymes |
| HMDH | 3CCW | Training | Other Enzymes |
| HS90A | 1UYG | Training | Miscellaneous |
| HXK4 | 3F9M | Training | Other Enzymes |

| | | | |
|---|---|---|---|
| IGF1R | 2OJ9 | Training | Kinase |
| ITAL | 2ICA | Training | Miscellaneous |
| JAK2 | 3LPB | Training | Kinase |
| KIT | 3G0E | Training | Kinase |
| KITH | 2B8T | Training | Other Enzymes |
| LKHA4 | 3CHP | Training | Protease |
| MAPK2 | 3M2W | Training | Kinase |
| MET | 3LQ8 | Training | Kinase |
| MK01 | 2OJG | Training | Kinase |
| MK10 | 2ZDT | Training | Kinase |
| MK14 | 2QD9 | Training | Kinase |
| MP2K1 | 3EQH | Training | Kinase |
| NRAM | 1B9V | Training | Other Enzymes |
| PARP1 | 3L3M | Training | Other Enzymes |
| PGH1 | 2OYU | Training | Other Enzymes |
| PGH2 | 3LN1 | Training | Other Enzymes |
| PLK1 | 2OWB | Training | Kinase |
| PPARA | 2P54 | Training | Nuclear Receptor |
| PPARD | 2ZNP | Training | Nuclear Receptor |
| PPARG | 2GTK | Training | Nuclear Receptor |
| PRGR | 3KBA | Training | Nuclear Receptor |
| PTN1 | 2AZR | Training | Other Enzymes |
| PUR2 | 1NJS | Training | Other Enzymes |
| PYRD | 1D3G | Training | Other Enzymes |
| RENI | 3G6Z | Training | Protease |
| ROCK1 | 2ETR | Training | Kinase |
| SAHH | 1LI4 | Training | Other Enzymes |
| SRC | 3EL8 | Training | Kinase |
| TGFR1 | 3HMM | Training | Kinase |
| THRB | 1YPE | Training | Protease |
| TRYB1 | 2ZEC | Training | Protease |
| UROK | 1SQT | Training | Protease |
| VGFR2 | 2P2I | Training | Kinase |
| WEE1 | 3BIZ | Training | Kinase |
| AMPC | 1L2S | Test | Other Enzymes |
| AOFB | 1S3B | Test | Other Enzymes |
| BACE1 | 3L5D | Test | Protease |
| DHI1 | 3FRJ | Test | Other Enzymes |
| DPP4 | 2I78 | Test | Protease |
| FNTA | '3E37 | Test | Other Enzymes |
| INHA | 4TRJ | Test | Other Enzymes |
| KIF11 | 3CJO | Test | Miscellaneous |
| PA2GA | 1KVO | Test | Other Enzymes |

| | | | |
|---|---|---|---|
| PDE5A | 1UDT | Test | Other Enzymes |
| PNPH | 3BGS | Test | Other Enzymes |
| XIAP | 3HL5 | Test | Miscellaneous |

## Table S2. Top 24 compounds selected by combining ExptGMS and GlideSP

| Cpd. ID | SMILES | Inhibition Rate ([Cpd.]=40 uM) | $IC_{50}$(uM) | Docking Score | ExptGMS |
|---|---|---|---|---|---|
| E1 | Cn1c(NC(=O)c2cnn3c(C(F)(F)F)cc(-c4cccc5ccccc45)nc23)nc2ccccc21 | 0.82 | 1.9 | -9.06 | 21169.39 |
| E2 | CCn1c(=O)n(CCC(=O)NC(Cc2ccccc2)c2nc3ccccc3[nH]2)c2ccccc21 | 0.67 | 13.6 | -9.08 | 21311.08 |
| E3 | CC(=O)N1CCc2c(sc3c2c(=O)n(-c2ccc(F)cc2)c(=O)n3Cc2cc(C)ccc2C)C1 | 0.92 | 5.7 | -9.09 | 21668.08 |
| E4 | O=C1C(=CNN(Cc2ccccc2)c2ccccc2)c2ccccc2C(=O)N1c1cccc(Cl)c1 | 0.65 | 21.2 | -9.45 | 21426.49 |
| E5 | CC(=O)N1CCc2c(sc3c2c(=O)n(-c2ccccc2)c(=O)n3Cc2cc(C)ccc2C)C1 | 0.64 | >100 | -9.18 | 21469.62 |
| E6 | O=C(c1cccnc1)N1CCc2c([nH]c3ccccc23)C1c1cccc(C(F)(F)F)c1 | 0.55 | >100 | -9.64 | 20892.34 |
| E7 | CC(=O)N1C(C(=O)N(CCc2ccccc2)Cc2ccccc2)CSC1c1ccccc1O | 0.63 | >100 | -9.08 | 21364.55 |
| E8 | Cc1cccc(Cn2c(=O)c(C(=O)NCc3ccco3)nn(-c3ccccc3F)c2=O)c1 | 0.66 | >100 | -9.00 | 21183.94 |
| E9 | CCc1ccccc1N(CC(=O)Nc1ccccc1C(=O)NC(C)CC)S(=O)(=O)c1ccccc1 | 0.54 | >100 | -9.05 | 21546.85 |
| E10 | COc1ccc(/N=C(/Nc2ccc(OC)cc2)c2cccc3ccccc23)cc1 | <0.5 | Not tested | -9.03 | 20870.44 |
| E11 | CN(CCCN(Cc1cccn1Cc1ccc(Cl)cc1)C(=O)Nc1ccccc1)c1ccccc1 | <0.5 | Not tested | -9.27 | 20958.15 |
| E12 | CCOc1ccc(N(Cc2nnc3n2CCCCC3)C(=O)Nc2ccc(C)cc2)cc1 | <0.5 | Not tested | -9.53 | 21167.00 |
| E13 | CC(=O)N1CCc2c(sc3c2c(=O)n(Cc2ccccc2)c(=O)n3Cc2cccc(C)c2)C1 | <0.5 | Not tested | -9.62 | 21201.15 |
| E14 | COc1cccc(-n2c(=O)c3c(C)c(C(=O)N(C)C)sc3n(Cc3cccc(F)c3)c2=O)c1 | <0.5 | Not tested | -9.06 | 21202.06 |
| E15 | CCN(CC)C(=O)c1sc2c(c1C)c(=O)n(-c1ccccc1)c(=O)n2Cc1cccc(Cl)c1 | <0.5 | Not tested | -9.20 | 21228.56 |
| E16 | CC(=O)N1CCc2c(sc3c2c(=O)n(-c2ccccc2C)c(=O)n3Cc2cc(C)ccc2C)C1 | <0.5 | Not tested | -9.24 | 21233.53 |
| E17 | NC(=O)C[C@H]1CN(C(=O)COc2ncccn2)C[C@H]1OC(=O)c1ccccc1 | <0.5 | Not tested | -8.98 | 21236.66 |
| E18 | CC(=O)c1ccc(C(=O)N2CCc3c([nH]c4ccccc34)C2c2ccccc2F)s1 | <0.5 | Not tested | -9.24 | 21251.32 |
| E19 | COc1cccc(CN(CCc2c[nH]c3ccccc23)C(=O)c2ccnn2C)c1 | <0.5 | Not tested | -9.23 | 21288.68 |
| E20 | COCc1ccc(C(=O)N(Cc2ccccc2)Cc2nc3ccccc3c(=O)[nH]2)cc1 | <0.5 | Not tested | -9.08 | 21469.89 |
| E21 | O=C1[C@@H]2Cc3c([nH]c4ccccc34)C(c3cccc([N+](=O)[O-])c3)N2C(=O)CN1CC(O)c1ccccc1 | <0.5 | Not tested | -9.39 | 21479.44 |
| E22 | O=C(c1cc(=O)c2ccccc2o1)N1CCc2c([nH]c3ccccc23)C1c1ccccc1F | <0.5 | Not tested | -9.34 | 21562.80 |
| E23 | O=C(CSc1nc(-c2ccccc2F)nc2c1cnn2-c1ccccc1)NC1CC1 | <0.5 | Not tested | -9.20 | 21594.12 |
| E24 | COc1ccc(N2C(=O)C3C(Cc4c[nH]c5ccccc45)NC4(C(=O)Nc5ccccc54)C3C2=O)cc1 | <0.5 | Not tested | -9.21 | 21597.12 |

Notes: Inhibition rate and $IC_{50}$ for each compound were determined by triplicate tests.

## Table S3. Top 24 compounds selected by GlideSP.

| Cpd. ID | SMILES | Inhibition Rate ([Cpd.]=40 uM) | $IC_{50}$(uM) | Docking Score | ExptGMS |
|---|---|---|---|---|---|
| D1 | C/C(=N\Nc1nc2c(c(=O)[nH]c(=O)n2C)n1Cc1cccc2ccccc12)c1ccc(O)cc1 | 1.49 | 11.8 | -9.86 | 22731.12 |
| D2* | O=C(CN1C(=O)NC2(CCCC2)C1=O)Nc1ccccc1C(=O)Nc1ccc2c(c1)OCO2 | 0.56 | >100 | -9.83 | 23155.50 |

| | | | | | |
|---|---|---|---|---|---|---|
| D3 | Cc1c(C(=O)NCCC2=CCCCC2)sc2ncnc(N(C)c3ccccc3)c12 | 0.63 | >100 | -10.27 | 23245.57 |
| D4 | COc1ccc(Cn2cc(C(CC(=O)N3CCCC3)c3ccccc3F)c3ccccc32)cc1 | <0.5 | Not tested | -10.31 | 21693.08 |
| D5 | COc1ccccc1-n1c(=O)c2c3c(sc2n(Cc2cccc(F)c2)c1=O)CN(C(C)=O)CC3 | <0.5 | Not tested | -10.15 | 21806.66 |
| D6 | CC(C)C(NC(=O)c1ccccc1Cl)C(=O)OCC(=O)Nc1cccc(S(N)(=O)=O)c1 | <0.5 | Not tested | -10.14 | 22088.40 |
| D7 | COc1ccccc1-n1c(=O)c2c3c(sc2n(Cc2cccc(Cl)c2)c1=O)CN(C(C)=O)CC3 | <0.5 | Not tested | -9.94 | 21785.43 |
| D8 | O=C(c1ccsc1)N1CCC[C@H](n2c(=O)n(Cc3cccc(F)c3)c3cccnc32)C1 | <0.5 | Not tested | -9.94 | 22080.41 |
| D9 | Cc1ccccc1N1C(=O)C(=Cc2ccc(N3CCOCC3)cc2)c2ccccc2C1=O | <0.5 | Not tested | -9.86 | 22733.06 |
| D10 | C/C(=N\Nc1nc2c(c(O)nc(=O)n2C)n1Cc1cccc2ccccc12)c1ccc(O)cc1 | <0.5 | Not tested | -9.84 | 22681.95 |
| D11 | Cc1ccc(N2C(=O)N(CC(=O)Nc3cccc(S(N)(=O)=O)c3)C(=O)C2C)cc1 | <0.5 | Not tested | -9.83 | 23156.32 |
| D12 | CC1CC(C)CN(C(=O)c2ccccc2NC(=O)N2CC(O)CC(F)(F)C2)C1 | <0.5 | Not tested | -9.71 | 23117.13 |
| D13 | CC(C)[C@H](NS(=O)(=O)c1ccccc1F)C(=O)OCC(=O)Nc1cccc(S(N)(=O)=O)c1 | <0.5 | Not tested | -9.70 | 22572.58 |
| D14 | Cc1c(C(=O)N(C)Cc2ccccc2)sc2ncnc(N(C)c3ccccc3)c12 | <0.5 | Not tested | -9.69 | 23754.33 |
| D15 | Cn1nccc1C(=O)N1CCC[C@H](n2c(=O)n(Cc3ccccc3)c3cccnc32)C1 | <0.5 | Not tested | -9.69 | 22216.58 |
| D16 | O=CN1CCCN(C(=O)CC2C(=O)NCCN2Cc2cccc(Oc3ccccc3)c2)CC1 | <0.5 | Not tested | -9.69 | 22979.45 |
| D17 | COc1ccccc1-n1c(=O)c2c3c(sc2n(Cc2ccccc2)c1=O)CN(C(C)=O)CC3 | <0.5 | Not tested | -9.69 | 21693.48 |
| D18 | O=C(c1ccco1)N1CCC[C@H](n2c(=O)n(Cc3ccccc3)c3cccnc32)C1 | <0.5 | Not tested | -9.68 | 22220.92 |
| D19 | O=C(Nc1ccccc1)N1CCCC(C(=O)N2CC(C(=O)N3CCCCC3)Oc3ccccc32)C1 | <0.5 | Not tested | -9.68 | 23174.05 |
| D20 | C[C@@]1(O)C[C@H](O)CN(C(=O)[C@@H](N)Cc2ccc(O)cc2)C[C@H]1n1cnc2c(N)nc(N)nc21 | <0.5 | Not tested | -9.68 | 23238.45 |
| D21 | O=C(NC(c1ccccc1)c1ccc2[nH]cnc2c1)C1=NN(C2CCS(=O)(=O)C2)C(=O)CC1 | <0.5 | Not tested | -9.66 | 22390.95 |
| D22 | CC(=O)N1CCc2c(sc3c2c(=O)n(-c2ccccc2)c(=O)n3CC(=O)c2ccc(F)cc2)C1 | <0.5 | Not tested | -9.66 | 21977.97 |
| D23 | COCCn1/c(=N/C(=O)c2c(F)cccc2F)sc2cc(S(N)(=O)=O)ccc21 | <0.5 | Not tested | -9.65 | 22580.34 |
| D24 | O=C(c1cccc(O)c1)N1C[C@@H](c2cc(F)cc(F)c2)[C@@H]2[C@H]1C1CCN2CC1 | <0.5 | Not tested | -9.64 | 22267.34 |

Notes: Inhibition rate and $IC_{50}$ for each compound were determined by triplicate tests. *For Cpd. D2, six replicate tests were performed.

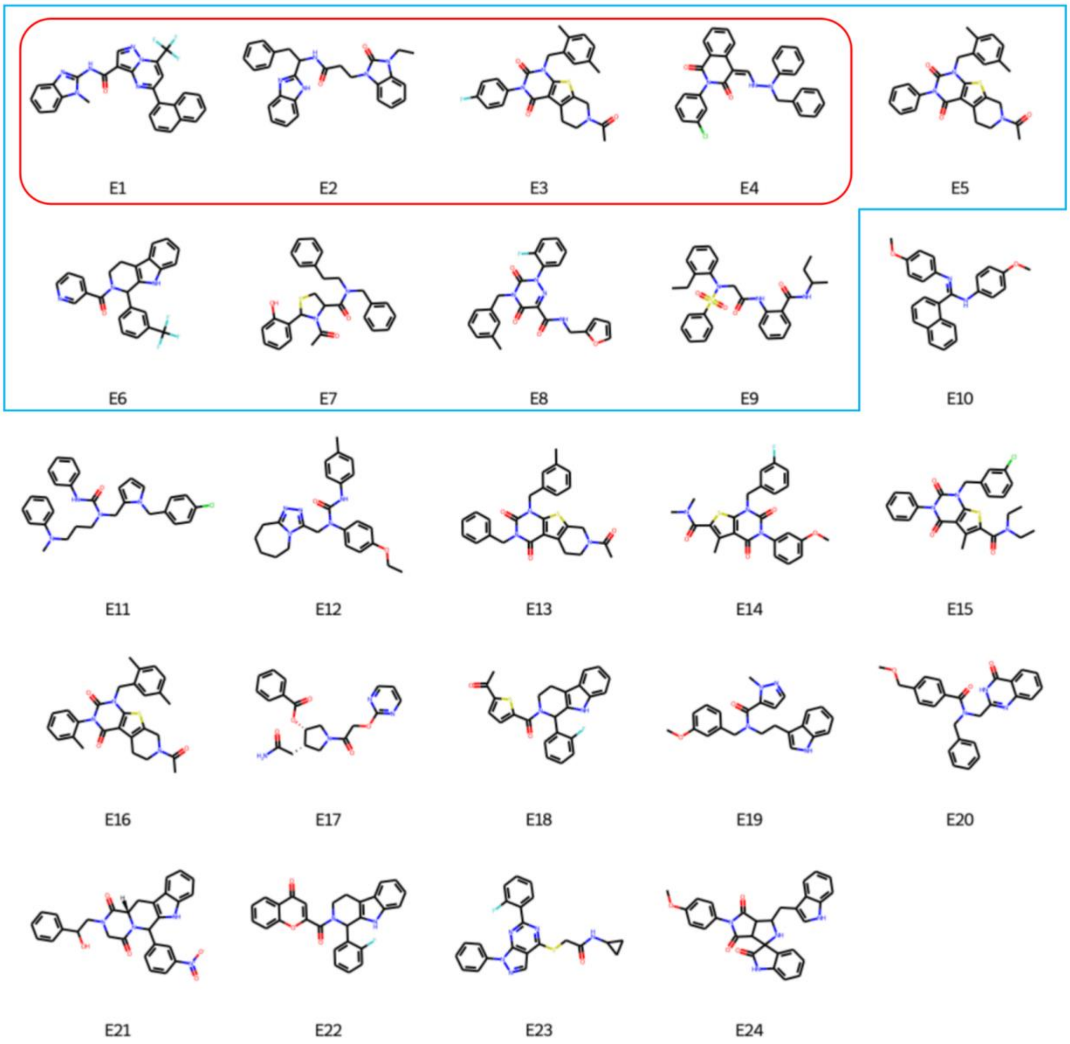


Figure S2. *Structures of top 24 compounds selected by ExptGMS+GlideSP*. Molecules with inhibition rates greater than 50% are indicated with a blue box. Molecules displayed in figure 6 are indicated with a red box.

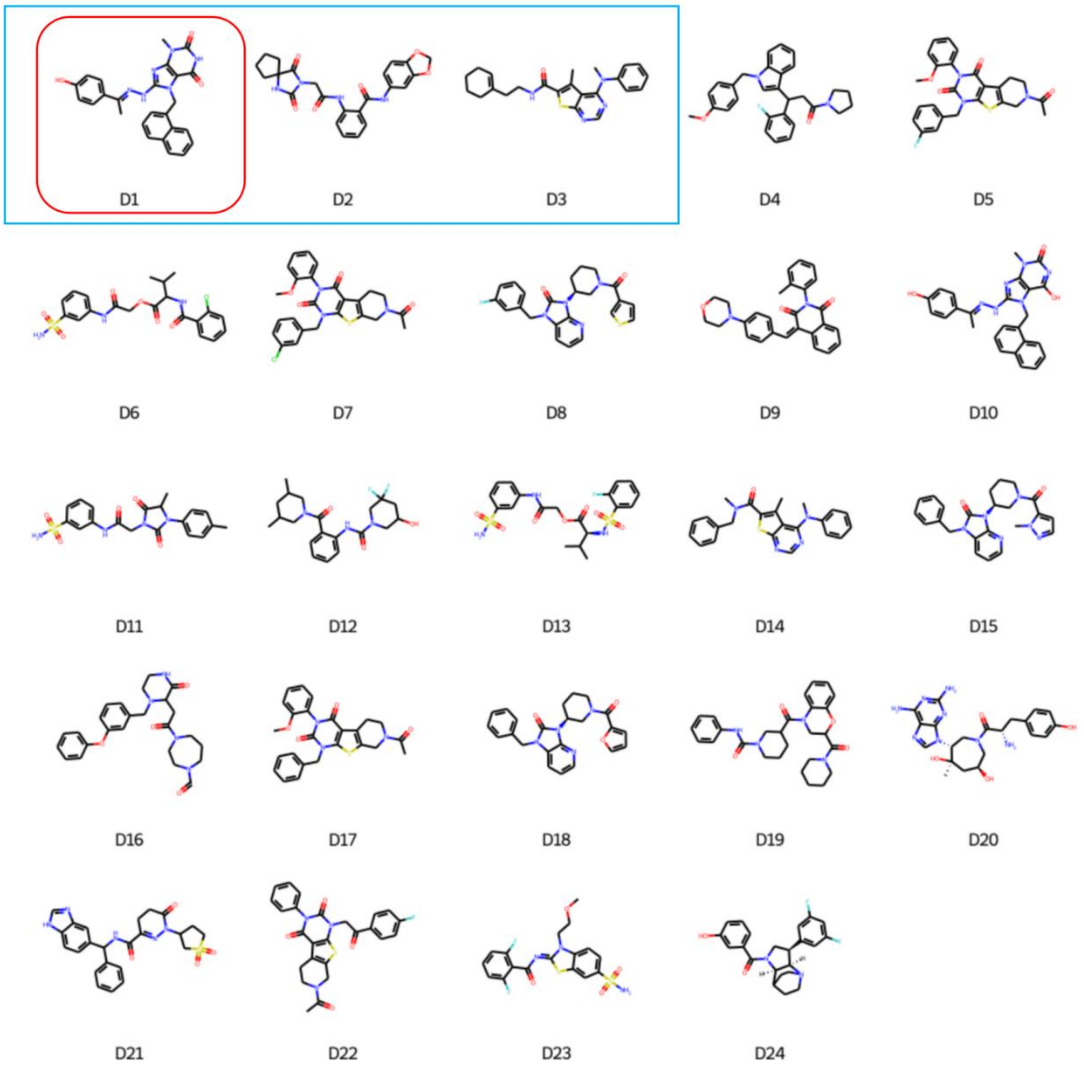


Figure S3. *Structures of top 24 compounds selected by GlideSP*. Molecules with inhibition rates greater than 50% are indicated with a blue box. Molecules displayed in figure 6 are indicated with a red box.

**Table S4. Details of GBDT training parameters**

| Key Parameters | Other Parameters |
|---|---|
| max_iter = 500<br>class_weight='balanced'<br>interaction_cst is restraint such that ED with different resolution will not interact with each other, with details listed below:<br>'ds_ed_only': {'cols': ['normed_glide_score',<br>'normed_ed25',<br>'normed_ed30',<br>'normed_ed35',<br>'normed_ed45',<br>'normed_ed55',<br>],<br>'interaction_cst': [set(comb) for comb in itertools.product([0], list(range(1, 6)))]<br>},<br>'ds_tf3p': {'cols': ['normed_glide_score',<br>'normed_tf3p'],<br>'interaction_cst': [{0, 1}]<br>},<br>'ds_mmgbsa': {'cols': ['normed_glide_score',<br>'normed_mmgbsa'],<br>'interaction_cst': [{0, 1}]<br>},<br>'ds_fpocket': {'cols': ['normed_glide_score',<br>'normed_fpocket_ed'<br>],<br>'interaction_cst': [{0, 1}]<br>},<br>'ds_usrcat': {'cols': ['normed_glide_score',<br>'usrcat'<br>],<br>'interaction_cst': [{0, 1}]<br>}, | learning_rate = 0.1<br>max_leaf_nodes = 31<br>max_depth=None<br>min_samples_leaf=20<br>l2_regularization=0<br>max_bins=255<br>warm_start=False<br>n_iter_no_change=10<br>tol=1e-7 |

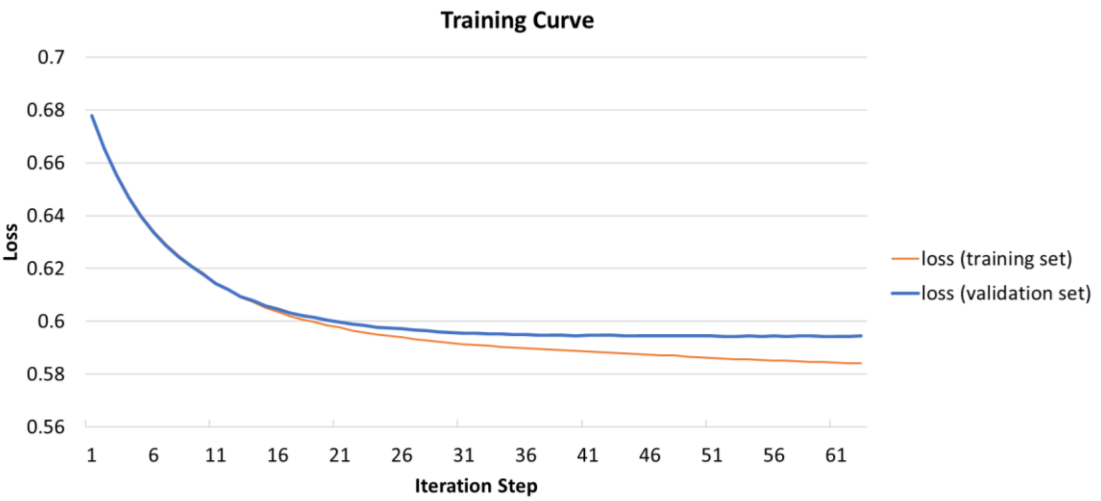


Figure S4. *Training curve of GBDT (GlideSP and multi-resolution ExptGMS).* The performance of

GBDT on the training set is shown through a plot showing the loss versus iterations. To prevent overfitting, a validation set comprising 10% of the training data was created. It is important to note that the data from the validation set were not used during the training process, but rather reserved for the purpose of early stopping to mitigate overfitting. The training was initially set to iterate 500 steps. As shown in the above figure, it stopped at step 63. During the training process, it is observed that the validation loss initially decreases and subsequently stabilizes, without showing signs of an upward trend. This pattern suggests the absence of overfitting in the model.
