# Supplementary material for: Using macromolecular electron densities to improve the enrichment of active compounds in virtual screening
Source: Commun Chem. 2023 Aug 22;6:173. doi: 10.1038/s42004-023-00984-5 (PMC10444862; doi:10.1038/s42004-023-00984-5)
Supplement: Supplementary file 3 — Description of Additional Supplementary Files [file 42004_2023_984_MOESM3_ESM.pdf]

Description of Additional Supplementary Files

File name: Supplementary Data 1
Description: Top N enrichment by target in DUD-E (details for Figure 2)

File name: Supplementary Data 2
Description: Top N enrichment by target in test set (details for Table 1)

File name: Supplementary Data 3
Description: ExptGMS scores and GBDT probability for each compound in test set (full data for Table 1)
